# Three-dimensional mandibular characteristics in skeletal malocclusion: A cross-sectional study

**DOI:** 10.1007/s00056-022-00419-1

**Published:** 2022-08-26

**Authors:** Carolin Olbrisch, Petra Santander, Norman Moser, Daniela Klenke, Philipp Meyer-Marcotty, Anja Quast

**Affiliations:** 1 Department of Orthodontics, University Medical Center Goettingen, Robert-Koch-Str. 40, 37075 Goettingen, Germany; 2 Department of Oral and Maxillofacial Surgery, University Medical Center Goettingen, Robert-Koch-Str. 40, 37075 Goettingen, Germany; 3 Department of Orthodontics, University of Marburg, Georg-Voigt-Str. 3, 35039 Marburg, Germany

**Keywords:** Adults, Skeletal pattern, Mandible, 3D cephalometry, Cone-beam computed tomography, Erwachsene, Skelettale Muster, Mandibula, 3‑D-Kephalometrie, Digitale Volumentomographie

## Abstract

**Purpose:**

We aimed to comprehensively analyse a possible correlation between skeletal malocclusions, gender and mandibular characteristics in all three dimensions in adults and to identify mandibular characteristics that are typical for extreme skeletal patterns.

**Methods:**

A 3D model of the skull was calculated in 111 adult patients (mean age = 27.0 ± 10.2 years; 49 women, 62 men) from available computed tomography or cone beam computed tomography scans of their heads. Based on the 3D models, the skeletal patterns were examined in (a) the transversal dimension regarding asymmetry according to menton deviation, (b) the sagittal dimension according to the Wits appraisal and (c) the vertical dimension according to the maxillomandibular plane angle. The mandibular characteristics assessed were linear (ramus height and width, body length), angular (ramus, gonial and body angle) and volumetric (ramus/mandibular volume, body/mandibular volume) parameters.

**Results:**

No correlation between transversal skeletal asymmetry and mandibular characteristics were found, while sagittal (*F*(16, 174) = 3.32, *p* < 0.001, η^2^ = 0.23) and vertical (*F*(16, 174) = 3.18, *p* < 0.001, η^2^ = 0.23) skeletal patterns were shown to have a significant effect on the mandible. Gender correlated with mandibular characteristics independently from the skeletal pattern. Discriminant analysis revealed that class II and III patients differed in ramus and body angle with class II patients showing higher angles (ramus angle: class II = 89.8 ± 3.9° vs. class III = 84.4 ± 4.8°; body angle: class II = 87.7 ± 4.8° vs. class III = 82.1 ± 5.2°). Hypo- and hyperdivergent patients were discriminated by gonial angle, body angle and body/mandibular volume with hyperdivergent patients having a greater gonial and body angle and body/mandibular volume (gonial angle: hypodivergent = 114 ± 9.3° vs. hyperdivergent = 126.4 ± 8.6°; body angle: hypodivergent = 82.9 ± 4.4° vs. hyperdivergent = 87.7 ± 6.5°; body/mandibular volume: hypodivergent = 72.4 ± 2.7% vs. hyperdivergent = 76.2 ± 2.6%).

**Conclusion:**

When analysing 3D data for treatment planning of adult patients, the orthodontist should pay attention to angular and volumetric characteristics of the mandible to identify extreme skeletal sagittal or vertical malocclusions.

**Supplementary Information:**

The online version of this article (10.1007/s00056-022-00419-1) contains supplementary material, which is available to authorized users.

## Abstract (de)

**Zielsetzung:**

Das Ziel der vorliegenden Studie lag in einer umfassenden dreidimensionalen Analyse einer möglichen Korrelation zwischen skelettalen Malokklusionen, dem Geschlecht und mandibulären Charakteristika bei Erwachsenen und der Identifizierung von Mandibulacharakteristika, welche typischerweise bei extremen Gesichtsschädelaufbauten auftreten.

**Methoden:**

Von 111 erwachsenen Patienten (mittleres Alter = 27,0 ± 10,2 Jahre; 49 Frauen, 62 Männer), bei denen ein Computertomographie- oder ein digitaler Volumentomographie-Scan des Kopfes vorlag, wurde ein 3‑D-Modell des Schädels erstellt. Anhand dessen wurden der Gesichtsschädelaufbau in a) der transversalen Dimension bezüglich möglicher Asymmetrien anhand der Menton-Deviation, b) der sagittalen Dimension anhand des Wits-Wertes und c) der vertikalen Dimension anhand des Interbasiswinkels klassifiziert. Die Mandibulacharakteristika wurden linear (Ramushöhe und -breite, Corpuslänge), angulär (Ramus, Kiefer- und Corpuswinkel) und volumetrisch (Ramus/Mandibula-Volumen, Corpus/Mandibula-Volumen) beurteilt.

**Ergebnisse:**

Es wurde kein Zusammenhang zwischen einer transversalen Gesichtsasymmetrie und den Mandibulacharakteristika gefunden. Jedoch zeigte sich, dass der sagittale (*F*(16, 174) = 3,32, *p* < 0,001, η^2^ = 0,23) und vertikale (*F*(16, 174) = 3,18, *p* < 0,001, η^2^ = 0,23) Gesichtsschädelaufbau einen signifikanten Einfluss auf die Mandibula hatte. Das Geschlecht beeinflusste unabhängig vom Gesichtsschädelaufbau die Charakteristika der Mandibula.

Klasse-II- und –III-Patienten unterschieden sich signifikant bezüglich Ramus- und Corpuswinkel (Ramuswinkel: Klasse II = 89,8 ± 3,9° vs. Klasse III = 84,4 ± 4,8°; Corpuswinkel: Klasse II = 87,7 ± 4,8° vs. Klasse III = 82,1 ± 5,2°). Patienten mit skelettal offenem und tiefem Biss unterschieden sich signifikant hinsichtlich Kieferwinkel, Corpuswinkel und Corpus/Mandibula-Volumen (Kieferwinkel: Tiefbiss = 114 ± 9,3° vs. offener Biss = 126,4 ± 8,6°; Corpuswinkel: Tiefbiss = 82,9 ± 4,4° vs. offener Biss = 87,7 ± 6,5°; Corpus/Mandibula-Volumen: Tiefbiss = 72,4 ± 2,7 % vs. offener Biss = 76,2 ± 2,6 %),

**Schlussfolgerung:**

Um extreme sagittale oder vertikale Dysgnathien zu erkennen, sollte der Kieferorthopäde bei der Analyse von 3‑D-Daten für die Behandlungsplanung erwachsener Patienten auf anguläre und volumetrische Charakteristika der Mandibula achten.

## Introduction

Predicting the skeletal growth pattern of children and adolescents is essential in orthodontic diagnosis and treatment [1, 2]. To develop an adequate treatment plan, the clinician has to estimate the craniofacial growth tendencies correctly. In particular, extreme growth patterns that require combined orthodontic and surgical treatment should be identified early to avoid burdening the young patient with ineffective and long-lasting therapies.

Unfortunately, growth and the development of skeletal malocclusions have uncertainties since the regulation of craniofacial growth is influenced by multiple genetic [3], environmental and functional factors [4]. Most clinicians rely on their experience and knowledge from previous research on two-dimensional (2D) lateral cephalograms, e.g. Björk’s growth studies [5–7], when estimating patients’ growth tendencies. However, information in 2D radiographs is limited as skeletal characteristics cannot be assessed in the transversal dimension and, even in combination with frontal 2D cephalograms, not all relevant information can be obtained. Furthermore, distortion, magnification and overlaps occur [8]. Although three-dimensional (3D) radiological imaging, like 2D radiographs, is only a snapshot of the dynamic growth process, it provides comprehensive, more detailed and more accurate information about bone morphology [8]. Therefore, it could potentially help to identify further skeletal characteristics that indicate an evolving extreme skeletal pattern. Though 3D imaging by cone-beam computed tomography (CBCT) or computed tomography (CT) is not yet part of routine orthodontic diagnostics [9], it has become increasingly popular in orthodontics. Hence, if a CBCT or CT is performed because of special indications [10], it could be analysed comprehensively and as much information as possible should be obtained.

But what skeletal parameters should the clinician pay attention to? One way to deal with this complex question is to understand the correlation between skeletal patterns and severe malocclusions in adults with the indication for orthognathic surgery. Looking at these particular patients enables us to identify skeletal characteristics, which typically accompany skeletal deformities [11].

Of all craniofacial features, the mandibular characteristics are of special interest [7, 12]. Up to now, most 3D studies focused on the correlation between facial asymmetry, i.e. transversal skeletal patterns, and mandibular characteristics in adult patients [13–16]. It has also been shown that gender can influence the expression of mandibular characteristics [17–19]. The relationship between mandibular characteristics and skeletal malocclusions in the sagittal and vertical dimension has attracted minor attention [17, 20], although this has been of great interest in the past using 2D cephalometric analysis [7]. Therefore, the aim of our study was to eliminate this lack of knowledge by comprehensively analysing the correlation between skeletal malocclusions, gender and mandibular characteristics in adults in all three dimensions. We hypothesized that the linear (ramus height, ramus width, body length), angular (ramus angle, gonial angle, body angle) and volumetric (ramus/mandibular volume, body/mandibular volume) mandibular characteristics correlate with skeletal classes, vertical relations, degrees of asymmetry and gender.

## Materials and methods

### Subjects

A total of 111 adult patients of the Department of Orthodontics and/or the Department of Oral and Maxillofacial surgery at the University Medical Center Goettingen with available CT or CBCT scans of their head were recruited for this cross-sectional study. The scans were performed in 94 patients for virtual orthognathic surgery planning and in 17 patients because of suspected midface trauma, inflammatory processes or traumatic brain injury between 2016 and 2019. The inclusion criteria for this study were the availability of a complete CT/CBCT scan of the viscerocranium and the anterior cranial base in sufficient quality and bilateral occlusal support in the molar region. Exclusion criteria were as follows: fractures of the viscerocranium except the midface, head and neck oncological diseases, craniofacial malformations such as cleft lip and palate, congenital jaw anomalies or previous orthognathic surgery.

All patients were informed about the procedure of the study and gave written informed consent for participation and use of their data. In accordance with the declaration of Helsinki, the ethics committee of the institution approved the study under application number 7/1/16.

### Three-dimensional modelling

The head CT scans (Spiral Scan, Somatom Definition AS, Siemens Healthcare GmbH, Erlangen, Germany; 128-line multidetector, layer thickness 0.6 mm) and head CBCT scans (PaX Zenith 3D, OrangeDental, Biberach an der Riss, Germany; field of view 240 × 190 mm, voxel size 0.3 mm) of the patients were exported in Digital Imaging and Communications in Medicine format (DICOM).

Using the software Mimics inPrint 3.0. (Materialise, Leuven, Belgium), 3D models of the skulls were created from the DICOM datasets, as described previously [11]. The reconstruction started with segmentation of the skull. For this purpose, the threshold range was set according to the grayscale of bone tissue. As the grayscale of the bone tissue differed between the CTs/CBCTs of the participants, the lower threshold had to be determined individually. It was set between −89 and 689 units. The upper threshold was always set to the highest recorded density value. To facilitate the analysis of the mandible, it was separated from the rest of the skull and the teeth were removed from the mandible above the alveolar bone. Finally, the surface of the 3D models was smoothed, and holes were filled up to a diameter of 4 mm. The 3D modelling was carried out by one trained examiner (C.O.).

### Measurements

The 3D analysis of the mandible was performed with the software ProPlan 3.0 (Materialise, Leuven, Belgium). After import, the 3D models of the head were aligned according to the Frankfurt horizontal (FH) plane. Landmarks were set on the reconstructed 3D models and their positions were verified two-dimensionally on the axial, coronal and sagittal layers of the CTs/CBCTs.

**Fig. 1:**
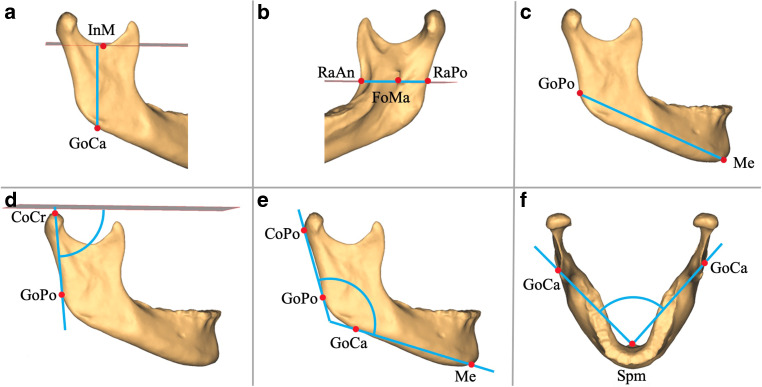
Illustration of the 3D measurements of the mandible. Linear measurements: **a** ramus height^a^: shortest distance between the most caudal point of the gonion (GoCa) and a plane parallel to Frankfurter horizontal (FH) plane passing through the most caudal point of the mandibular notch (InM); **b** ramus width^a^: shortest distance between the most anterior point of the ramus (RaAn) and the most posterior point of the ramus (RaPo) at the level of a plane parallel to FH plane passing through the foramen mandibulae (FoMa); **c** body length^a^: shortest distance between menton (Me) and the most posterior point of the gonion (GoPo). Angular measurements: **d** ramus angle^a^: angle between the FH plane and a connecting line through the most cranial point of the condylar head (CoCr) and the most posterior point of the gonion (GoPo), projected onto the midsagittal plane; **e** gonial angle^a^: angle between a connecting line through menton (Me) and the most caudal point of the gonion (GoCa) and a connecting line through the most posterior point of the condylar head (CoPo) and the most posterior point of the gonion (GoPo), projected onto the midsagittal plane; **f** body angle: angle between the most caudal point of the gonion right (GoCa), the most posterior point of spina mentalis (Spm) and the most caudal point of the gonion left (GoCa), projected onto the FH plane. ^a^measured bilaterally Veranschaulichung der 3‑D-Messungen der Mandibula. Lineare Messungen: **a** Ramushöhe^a^: kürzeste Distanz zwischen dem kaudalsten Punkt des Gonions (GoCa) und einer durch den kaudalsten Punkt der Incisura mandibulae (InM) und parallel zur FH verlaufenden Ebene; **b** Ramusbreite^a^: kürzeste Distanz zwischen dem am weitesten anterior gelegenen Punkt des Ramus (RaAn) und dem am weitesten posterior gelegenen Punkt des Ramus (RaPo) auf Höhe einer durch den Punkt Foramen mandibulae (FoMa) und parallel zur FH verlaufenden Ebene; **c** Corpuslänge^a^: kürzeste Distanz zwischen Menton (Me) und dem am weitesten posterior gelegenen Punkt des Gonions (GoPo). Anguläre Messungen: **d** Ramuswinkel^a^: Winkel zwischen einer Verbindungslinie durch den kranialsten Punkt des Condylus (CoCr) und den am weitesten posterior gelegenen Punkt des Gonions (GoPo) und der FH, auf die Median-Sagittalebene projiziert; **e** Kieferwinkel^a^: Winkel zwischen einer Verbindungslinie durch Menton (Me) und den kaudalsten Punkt des Gonions (GoCa) und einer Verbindungslinie durch den am weitesten posterior gelegenen Punkt des Condylus (CoPo) und den am weitesten posterior gelegenen Punkt des Gonions (GoPo), auf die Median-Sagittalebene projiziert; **f** Corpuswinkel: Winkel zwischen dem kaudalsten Punkt des Gonions (GoCa) rechts, Spina mentalis (Spm) und dem kaudalsten Punkt des Gonions links, auf die FH projiziert. ^a^Bilateral gemessen

**Fig. 2:**
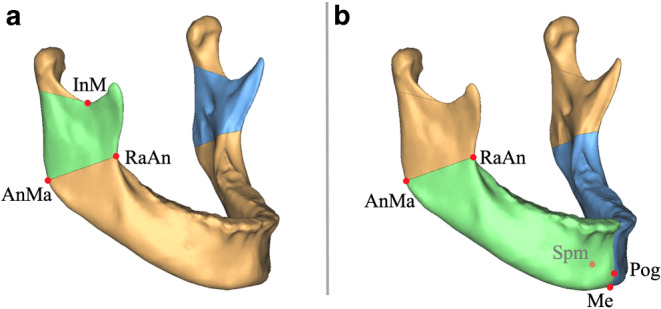
Illustration of the volumetric measurements of the mandible. **a** Ramus volume: cranial limited by a plane parallel to FH through the most caudal point of the mandibular notch (InM) and caudal limited by a plane through the most anterior point of the ramus (RaAn) and the most posteriocaudal point of the angulus mandibulae (AnMa) perpendicular to the mandibular surface; **b** body volume: posterior limited by a plane through the most anterior point of the ramus (RaAn) and the most posteriocaudal point of the angulus mandibulae (AnMa) perpendicular to the surface and anterior limited by a plane through menton (Me), pogonion (Pog) and the most posterior point of spina mentalis (Spm). Volumetric measurements were performed bilaterally (*green*, *blue*) Veranschaulichung der volumetrischen Messungen der Mandibula. **a** Ramusvolumen: kranial begrenzt durch eine Ebene parallel zur FH durch den kaudalsten Punkt der Incisura mandibulae (InM) und kaudal begrenzt durch eine Ebene durch den am weitesten anterior gelegenen Punkt des Ramus (RaAn) und den am weitesten posteriokaudal gelegenen Punkt des Angulus mandibulae (AnMa) senkrecht zur Oberfläche der Mandibula; **b** Corpusvolumen: posterior begrenzt durch eine Ebene durch den am weitesten anterior gelegenen Punkt des Ramus (RaAn) und den am weitesten posteriokaudal gelegenen Punkt des Angulus mandibulae (AnMa) senkrecht zur Oberfläche und anterior begrenzt durch eine Ebene durch Menton (Me), Pogonion (Pog) und den am weitesten posterior gelegenen Punkt der Spina mentalis (Spm). Die volumetrischen Messungen wurden bilateral durchgeführt (*grün*, *blau*)

Based on these landmarks mandibular characteristics were measured by eight linear, angular and volumetric variables (Figs. 1 and 2). The FH, frontal plane, midsagittal plane and occlusal plane were used as reference planes. FH was constructed by orbita on the right side and porion on both sides. Frontal plane was constructed by porion on both sides and perpendicular to FH. Midsagittal plane was constructed by nasion and perpendicular to FH and frontal plane. Occlusal plane was constructed by the mesiobuccal cusp tip of both upper first molars and the centre between the upper and lower incisal point.

To classify the skeletal malocclusion of the patients, the menton deviation (MD), the Wits appraisal and the maxillomandibular plane angle were assessed three-dimensionally. Menton deviation was defined as the shortest distance between menton and the midsagittal plane. It indicated the transversal skeletal pattern and divided the patients into symmetric (MD ≤ 2 mm), moderately asymmetric (MD = 2.1 to 4 mm) and severely asymmetric (MD > 4 mm) [21]. The Wits appraisal was defined as the shortest distance between the deepest point on the contour of the maxilla (point A) and the deepest point of the outline of the symphysis (point B), projected onto the occlusal plane and from there onto the midsagittal plane. It indicated the sagittal skeletal pattern and divided the patients into class I (Wits = −2 to 2 mm), class II (Wits > 2 mm) and class III (Wits < −2 mm) [22]. The maxillomandibular plane angle was defined as the angle between the mandibular plane (determined by menton and the most caudal point of gonion on both sides) and the nasal line (determined by spina nasalis anterior and spina nasalis posterior), projected onto the midsagittal plane. It indicated the vertical skeletal pattern and divided the patients into neutral (ML-NL = 20.5° to 26.5°), hypodivergent (ML-NL < 20.5°) and hyperdivergent (ML-NL > 26.5°) [23].

All measurements were performed by the same trained examiner, who performed 3D modelling (C.O.). In order to determine the intra- and interrater reliability ten 3D models were randomly selected. On these 3D models, the same examiner repeated all measurements after one year. In addition, another experienced examiner performed all measurements on these ten 3D models.

### Statistical analysis

Statistical analysis was performed using IBM SPSS Statistics for Macintosh 26.0 (IBM Corp., Armonk, NY, USA).

To investigate the correlation between the transversal skeletal pattern and the mandibular characteristics, the side differences of bilateral characteristics were calculated. For this purpose, the deviating side was subtracted from the nondeviating side. For the volumetric measurements, the side differences of the segmental volumes were set in relation to the total mandibular volume (segmental volume_non–dev_ − segmental volume_dev_) / total mandibular volume.

To investigate the correlation between the vertical and sagittal skeletal pattern and mandibular characteristics, the measurements of bilateral morphological characteristics were averaged. For the volumetric measurements, the segmental volumes of both sides were added and set in relation to the total mandibular volume (segmental volume_right_ + segmental volume_left_) / total mandibular volume.

Intra- and interrater reliability was investigated separately for each measurement by Bland–Altman plots [24]. Normal distribution of the measurements was confirmed by q–q plots. Unequal sample sizes between the groups were balanced using bootstrapping (1000 bootstrap samples, BCa [bias-corrected and accelerated] intervals). To examine whether age was a confounder, analysis of variance (ANOVA) was conducted. Means and standard deviations of the mandibular characteristics according to the skeletal pattern were reported. In the main analysis, a multivariate analysis of variance (MANOVA) was performed to analyse the independent and dependent effects of the degree of asymmetry and gender on side-specific mandibular characteristics. Another MANOVA was performed to analyse the independent and depended effects of the sagittal and vertical skeletal pattern and gender on the mandibular characteristics. The significance level was set at α = 0.05. Multicollinearity was excluded using Pearson’s correlation. The equality of variances was checked using Levene’s test. The F‑values and *p*-values were reported using Pillai’s trace. To identify mandibular characteristics that differ best between the skeletal patterns, stepwise discriminant analyses were performed [25]. Post hoc power analysis for “MANOVA: global effects” revealed a statistical power over 99% assuming a sample size of 111 patients in three groups (G*Power 3.1.9.2, University of Dusseldorf, Germany).

## Results

Demographic data and distribution of the skeletal patterns of the participants are presented in Table 1.

**Table 1:** Demographic data and skeletal pattern of the participants Demographische Daten und Gesichtsschädelaufbau der Studienteilnehmer

Patients	*N* = 111
Female	*n* = 49
Male	*n* = 62
*Patients’ age*	M = 27.0 years; SD = 10.2 years
*Transversal skeletal pattern (degree of asymmetry)*	
Symmetric (MD ≤ 2 mm)	*n* = 65 (M = 0.8 mm; SD = 0.6 mm)
Moderately asymmetric (MD = [2.1; 4 mm])	*n* = 22 (M = 2.9 mm; SD = 0.6 mm)
Strongly asymmetric (MD > 4 mm)	*n* = 24 (M = 6.2 mm; SD = 1.9 mm)
*Sagittal skeletal pattern*	
Class I (Wits = [−2; 2 mm])	*n* = 25 (M = −0.1 mm; SD = 1.2 mm)
Class II (Wits > 2 mm)	*n* = 36 (M = 6.5 mm; SD = 2.9 mm)
Class III (Wits < −2 mm)	*n* = 50 (M = −9.1 mm; SD = 4.3 mm)
*Vertical skeletal pattern*	
Neutral (ML-NL = [20.5; 26.5°])	*n* = 32 (M = 23.2°; SD = 1.8°)
Hypodivergent (ML-NL < 20.5°)	*n* = 37 (M = 16.3°; SD = 4.1°)
Hyperdivergent (ML-NL > 26.5°)	*n* = 42 (M = 32.7°; SD = 4.3°)

*MD* Menton deviation from the midsagittal plane; *ML-NL* Maxillomandibular plane angle, *M* mean, *SD* standard deviation

Age of the study population was equally distributed between the skeletal groups (*p* > 0.05), except between the sagittal groups. Class III patients were found to be significantly younger than class I and II patients (*p* = 0.001). Their average age was 23.1 years, while class I and II patients were 31 and 29.4 years old, respectively. The investigation of the reliability of the linear, angular and volumetric measurements by Bland–Altman plots revealed high intra- and interrater agreements (Supplemental Table 1).

### Correlation between transversal skeletal symmetry and mandibular characteristics

Means and standard deviations of the lateral differences of the mandibular characteristics according to the transversal skeletal symmetry are presented in Table 2. MANOVA indicated that the transversal degree of asymmetry had no significant effect on side-specific differences in the mandibular characteristics, *F*(14, 200) = 1.66, *p* = 0.066, η^2^ = 0.10. For this reason, no subsequent stepwise discriminant analysis was conducted.

**Table 2:** Means (M) and standard deviations (SD) of the side-differences of the mandibular characteristics according to the transversal skeletal pattern (degree of asymmetry) Mittelwerte (M) und Standardabweichungen (SD) der Seitendifferenzen der morphologischen Mandibulacharakteristika in Abhängigkeit von der transversalen Klassifikation des Gesichtsschädelaufbaus (Asymmetriegrad)

Characteristic	Symmetric (*n* = 65)		Moderately asymmetric (*n* = 22)		Strongly asymmetric (*n* = 24)	
	M	SD	M	SD	M	SD
Diff Ramus height (mm)	0.2	2.4	0.1	2.7	1.4	3.0
Diff Ramus width (mm)	−0.5	1.7	−0.4	1.6	0.1	1.6
Diff Body length (mm)	0.1	2.0	0.6	2.2	1.7	1.9
Diff Ramus angle (mm)	−0.4	2.4	−0.2	2.6	−1.1	3.3
Diff Gonial angle (°)	0.4	13.1	−1.2	5.1	3.8	15.5
Diff Ramus/Mand volume (°)	−0.1	0.9	−0.3	0.8	0.0	1.0
Diff Body/Mand volume (%)	−0.1	1.6	0.1	1.7	0.6	2.0

*Diff* side-difference, calculated as: nondeviated side minus deviated side; *Mand* mandibular

### Correlation between sagittal skeletal patterns and mandibular characteristics

The means and standard deviations of the mandibular characteristics according to the sagittal skeletal patterns are presented in Table 3.

**Table 3:** Means (M) and standard deviations (SD) of the mandibular characteristics according to the sagittal skeletal pattern Mittelwerte (M) und Standardabweichungen (SD) der Charakteristika der Mandibula in Abhängigkeit von der sagittalen Klassifikation des Gesichtsschädelaufbaus

Characteristic	Class I (*n* = 25)		Class II (*n* = 36)		Class III (*n* = 50)	
	M	SD	M	SD	M	SD
Ramus height (mm)	49.3	6.0	47.0	5.8	48.7	6.6
Ramus width (mm)	31.9	3.7	30.4	3.5	30.1	3.1
Body length (mm)	92.2	5.8	88.3	6.3	95.6	7.6
Ramus angle (°)	90.1	4.1	89.8	3.9	84.4	4.8
Gonial angle (°)	115.8	10.6	117.5	10.7	124.2	9.2
Body angle (°)	85.2	6.5	87.7	6.0	82.1	5.2
Ramus/Mand volume (%)	19.8	2.7	19.7	2.7	18.7	2.7
Body/Mand volume (%)	74.2	2.9	74.5	3.3	74.5	2.6

*Mand* mandibular. Bilateral mandibular characteristics were averaged, except the bilateral volumetric characteristics which were added.

MANOVA demonstrated that the sagittal skeletal pattern had a significant effect on mandibular characteristics with a large effect size, *F*(16, 174) = 3.32, *p* < 0.001, η^2^ = 0.23.

MANOVA was followed up with a stepwise discriminant analysis, which revealed two discriminant functions. Ramus height, ramus width, body length, gonial angle, ramus/mandibular volume and body/mandibular volume were removed from the model. The only relevant discriminators included in both functions were the ramus angle und the body angle (first function: explained 96.7% of the variance, canonical *R*^*2*^ = 0.38; second function: explained only 3.3% of the variance, canonical *R*^*2*^ = 0.02). In combination, the discriminant functions significantly differentiated the sagittal groups, Λ = 0.6, χ^2^(4) = 54.32, *p* < 0.001. The ramus angle loaded more highly on the first function, which differentiated skeletal class III patients from skeletal class II patients (Table 4). The body angle loaded more highly on the second function, which differentiated skeletal class I patients from skeletal class II and III patients. Using both functions, 62.2% of the investigated patients were classified in the correct sagittal group. In particular, class III patients were reliably identified. The classification of skeletal class I patients was insufficient: 84% were falsely classified as skeletal class II or class III (Table 5).

**Table 4:** Correlations (*r*) between the discriminant functions for sagittal skeletal pattern and the included mandibular characteristics Korrelationen (*r) *zwischen den Diskriminanzfunktionen zum sagittalen Gesichtsschädelaufbau und den einbezogenen Charakteristika der Mandibula

Discriminant variable	Discriminant function	
	1	2
Ramus angle	0.79	−0.61
Body angle	0.53	0.85

**Table 5:** Classification of the sagittal skeletal pattern using the included morphological characteristics: ramus angle and body angle Klassifizierung des sagittalen Gesichtsschädelaufbaus anhand der einbezogenen morphologischen Charakteristika: Ramuswinkel und Corpuswinkel

Original group	Group, patients were classified based on ramus and body angle			
	Class I	Class II	Class III	Total
Class I: *n* (%)	4 (16.0)	14 (56.0)	7 (28.0)	25 (100.0)
Class II: *n* (%)	2 (5.6)	22 (61.1)	12 (33.3)	36 (100.0)
Class III: *n* (%)	2 (4.0)	5 (10.0)	43 (86.0)	50 (100.0)

**Fig. 3:**
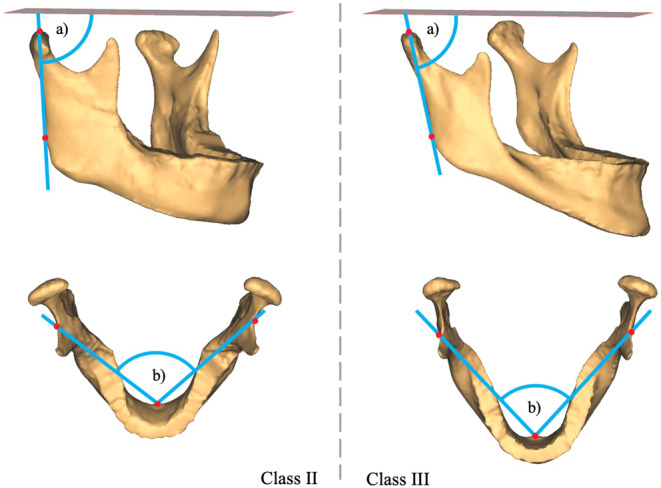
Characteristic mandible of a class II patient versus a class III patient. *a)* Ramus angle and *b)* body angle were found to be adequate discriminators of the sagittal skeletal pattern Charakteristische Mandibula, Klasse-II-Patient vs. Klasse-III-Patient. *a)* Ramuswinkel und *b)* Corpuswinkel erwiesen sich als geeignete Diskriminatoren des sagittalen Gesichtsschädelaufbaus

Fig. 3 shows characteristic mandibles of a class II and a class III patient from the study population. According to the means of the adequate discriminators for the sagittal skeletal pattern, class II patients had a greater ramus and body angle than class III patients (Table 3).

### Correlation between vertical skeletal patterns and mandibular characteristics

The means and standard deviations of the mandibular characteristics according to the vertical skeletal patterns are presented in Table 6.

**Table 6:** Means (M) und standard deviations (SD) of the mandibular characteristics according to the vertical skeletal pattern Mittelwerte (M) und Standardabweichungen (SD) der Charakteristika der Mandibula in Abhängigkeit von der vertikalen Klassifikation des Gesichtsschädelaufbaus

Characteristic	Neutral (*n* = 32)		Hypodivergent (*n* = 37)		Hyperdivergent (*n* = 42)	
	M	SD	M	SD	M	SD
Ramus height (mm)	47.7	5.1	51.0	7.1	46.3	5.4
Ramus width (mm)	30.7	2.9	32.1	3.8	29.3	2.9
Body length (mm)	93.4	7.0	93.5	8.7	90.9	6.6
Ramus angle (°)	86.8	5.4	87.9	4.8	87.5	5.3
Gonial angle (°)	119.0	10.2	114.0	9.3	126.4	8.6
Body angle (°)	82.7	6.2	82.9	4.4	87.7	6.5
Ramus/Mand volume (%)	19.0	2.1	21.0	2.8	18.0	2.2
Body/Mand volume (%)	74.5	1.8	72.4	2.7	76.2	2.6

*Mand* mandibular. Bilateral mandibular characteristics were averaged, except the bilateral volumetric characteristics which were added

MANOVA also revealed that the vertical skeletal pattern had a significant effect on mandibular morphology with a large effect size, *F*(16, 174) = 3.18, *p* < 0.001, η^2^ = 0.23.

The following discriminant analysis revealed two discriminant functions, which removed ramus height, ramus width, body length, ramus angle and ramus/mandibular volume from the model. Included in both functions were gonial angle, body angle and body/mandibular volume (first function explained 94.9% of the variance, canonical *R*^*2*^ = 0.44; second function explained 5.1% of the variance, *R*^*2*^ = 0.04). In combination, these discriminant functions significantly differentiated the vertical groups, Λ = 0.54, χ^2^(6) = 66.71, *p* < 0.001. The gonial angle and the body/mandibular volume loaded more highly on the first function, which discriminated hypodivergent from hyperdivergent patients. The body angle loaded more highly on the second function, which discriminated neutral patients from hypodivergent and hyperdivergent patients (Table 7). Using both functions, 63.1% of the investigated patients were classified in the correct vertical group. Particularly hypodivergent and hyperdivergent patients were reliably identified. The classification of neutral patients, however, was insufficient (Table 8).

**Table 7:** Correlations (*r*) between the discriminant function for vertical skeletal pattern and the included mandibular characteristics Korrelationen (*r) *zwischen der Diskriminanzfunktion zum vertikalen Gesichtsschädelaufbau und den einbezogenen Charakteristika der Mandibula

Discriminant variable	Discriminant function	
	1	2
Gonial angle	0.65	0.04
Body angle	0.42	0.85
Body/mandibular volume	0.74	−0.55

**Table 8:** Classification of the vertical skeletal pattern using the included morphological characteristics: gonial angle, body angle and body/mandibular volume Klassifizierung des vertikalen Gesichtsschädelaufbaus anhand der einbezogenen morphologischen Charakteristika: Kieferwinkel, Corpuswinkel und Corpus/Mandibula-Volumen

Original group	Group, patients were classified based on gonial angle, body angle and body/mandibular volume			
	Neutral	Hypodivergent	Hyperdivergent	Total
Neutral, *n* (%)	13 (40.6)	10 (31.3)	9 (28.1)	32 (100.0)
Hypodivergent, *n* (%)	6 (16.2)	*28 (75.7)*	3 (8.1)	37 (100.0)
Hyperdivergent, *n* (%)	8 (19.0)	5 (11.9)	*29 (69.0)*	42 (100.0)

**Fig. 4:**
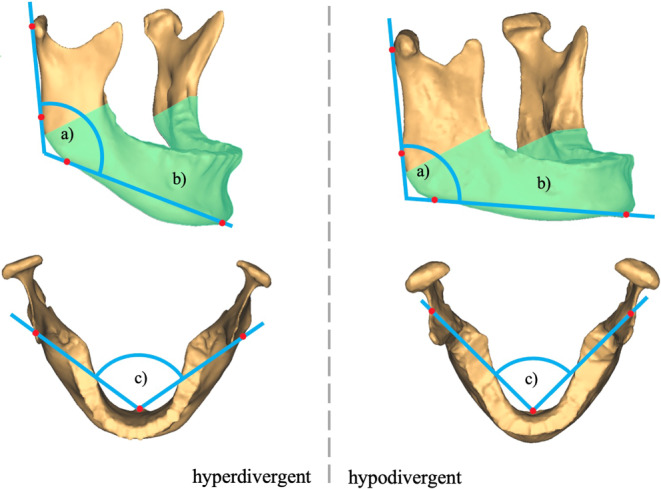
Characteristic mandible of a hyperdivergent patient versus a hypodivergent patient. *a)* Gonial angle, *b)* body angle and *c)* body/mandibular volume were found to be adequate discriminators of the vertical skeletal pattern Charakteristische Mandibula eines offenen Patienten vs. charakteristische Mandibula eines tiefen Patienten. *a)* Kieferwinkel, *b)* Corpuswinkel und *c)* Corpus/Mandibula-Volumen erwiesen sich als geeignete Diskriminatoren des vertikalen Gesichtsschädelaufbaus

Fig. 4 shows characteristic mandibles of a hyperdivergent and a hypodivergent patient from the study population. According to the means of the adequate discriminators for the vertical skeletal pattern, hyperdivergent patients showed a greater gonial and body angle and a greater body/mandible volume (Table 6).

MANOVA showed no significant interaction of sagittal and vertical skeletal patterns on mandibular characteristics, *F*(32, 356) = 0.68, *p* = 0.690.

### Correlation between gender, mandibular characteristics and skeletal patterns

MANOVA indicated that gender had no significant effect on side-specific differences in the mandibular characteristics, *F*(7, 99) = 0.63, *p* = 0.734, η^2^ = 0.042, and no significant interaction of the transversal skeletal pattern and gender on side-specific differences on the mandibular characteristics was revealed, *F*(14, 200) = 0.98, *p* = 0.474, η^2^ = 0.064.

MANOVA demonstrated that the gender had a significant effect on mandibular characteristics with a large effect size, *F*(8, 86) = 5.23, *p* < 0.001, η^2^ = 0.33. However, MANOVA showed no significant interaction of sagittal skeletal patterns and gender on mandibular characteristics, *F*(16, 174) = 1.16, *p* = 0.303, η^2^ = 0.10, and no significant interaction of vertical skeletal patterns and gender on mandibular characteristics, *F*(16, 174) = 0.68, *p* = 0.81, η^2^ = 0.07.

## Discussion

To the best of our knowledge, the present study is the first to provide comprehensive information on the correlation between mandibular characteristics, gender and skeletal patterns in all three dimensions. It demonstrates which mandibular characteristics seem to be typical for patients with severe skeletal malocclusions and gives a first hint which parameters could be paid special attention to when identifying a patient’s skeletal growth tendency using 3D imaging.

It was shown that the transversal skeletal symmetry pattern had no significant influence on mandibular characteristics. Analysing the correlation between mandibular characteristics and sagittal and vertical skeletal patterns revealed that sagittal and vertical skeletal patterns influenced mandibular characteristics independently from each other. While ramus and body angle correlated with the sagittal skeletal class, gonial angle, body angle and body/mandibular volume correlated with the vertical relation.

It was shown that gender had a significant influence on mandibular characteristics, but not on side-specific differences. However, gender and skeletal patterns influenced mandibular characteristics independently from each other.

### Mandibular characteristics related to transversal skeletal symmetry pattern

None of the mandibular characteristics correlated with the transversal skeletal symmetry pattern defined by menton deviation. This is contrary to our expectations, as other 3D studies had reported significantly greater side-differences in body length, ramus height and gonial angle in asymmetric patients than in symmetric patients [13–16, 26]. However, these studies did not distinguish between moderately asymmetric and severely asymmetric and simply used a menton deviation of 4 mm as the cut-off between symmetric and asymmetric patients. In addition, the groups of asymmetric patients in the cited studies had a greater menton deviation on average than the group of severely asymmetric patients in the present study [15–17].

### Mandibular characteristics related to sagittal skeletal pattern

To the authors’ knowledge, no study group has previously revealed a 3D correlation between sagittal skeletal patterns and the ramus or body angle. The few existing 3D studies on correlations between mandibular morphology and sagittal skeletal patterns mainly examined linear and volumetric mandibular characteristics [27–30]. However, our study showed that especially angular characteristics were related with sagittal skeletal patterns.

The ramus angle contributed essentially to the differentiation between class II and class III patients. Although ramus angle can already be assessed in daily practice using lateral cephalometrics, it is not part of standard cephalometric analysis [23]. Regardless of parameters such as distortion and magnification, clinical orthodontists when analysing sagittal skeletal patterns might henceforth examine it.

The evaluation of the body angle, possible with 3D imaging, provides new interesting information. Although it contributed more to the differentiation of class I patients from class II and III patients, it possibly gives valuable hints when assessing patients’ growth tendencies.

### Mandibular characteristics related to vertical skeletal pattern

Gonial angle, body angle and body/mandibular volume were found to correlate with the vertical skeletal pattern. Especially the gonial angle and the body/mandibular volume contributed to the differentiation between hypodivergent and hyperdivergent patients. A correlation between gonial angle and the vertical skeletal growth pattern was already discovered in the last century and has since been a basic element of established 2D cephalometric analyses, such as those of Jarabak and Fizzell [31], Björk [7] or Björk and Skieller [32]. As the present study has shown, the gonial angle also correlated with vertical skeletal patterns in the 3D analysis.

However, body angle and body/mandibular volume, which can only be analysed based on 3D data, have not been considered to correlate with vertical skeletal growth patterns so far. Previous 3D volumetric studies have only investigated the correlation between the total mandibular volume and vertical skeletal patterns and obtained inconsistent results [20, 33]: While Nakawaki et al. [20] reported a significantly larger total mandibular volume in hypodivergent than in hyperdivergent patients, Nair et al. [33] observed no significant correlation. In the present study, the correlation between the mandibular segment volume and vertical skeletal pattern was analysed for the first time to the authors’ knowledge. It was found that hypodivergent patients had a smaller body volume than hyperdivergent patients. Ramus volume tended to be larger in hypodivergent patients, but the characteristic did not prove to be an adequate discriminator of the vertical skeletal pattern (Table 6). Therefore, when 3D data are available, orthodontists might pay attention to differences in mandibular segment volume rather than to the total mandibular volume when trying to evaluate a patient’s vertical growth pattern.

### Limitations of the study

This study provided a reliable and objective method to analyse the mandible cephalometrically and volumetrically based on 3D data. However, the 3D measurement, especially of angles, is challenging, as pitch, roll and yaw of the reference structures can cause distortions [34]. Projecting the measured angle onto the nearest parallel reference plane, as performed in the herein study, proved to be a simple and sufficient method to avoid distortion [35].

Patients with fractures of the viscerocranium except the midface were excluded to avoid measurement bias due to dislocated fracture segments. Furthermore, patients with a midface fracture were only included if the original position of the landmarks orbita and spina nasalis anterior were still identifiable. It is unknown whether any orthodontic treatment was performed during the patients’ adolescence. Thus, possible therapeutic changes in growth patterns in the past might have caused a bias.

Distribution of age between the skeletal groups was analysed, as it is known to influence mandibular characteristics [36, 37]. Class III patients were significantly younger than class I and II patients in our sample. However, this age difference is negligible, as the average age of the sagittal groups differed by less than 10 years and differences in mandibular characteristics were only observed between larger age intervals [36].

As mentioned above, the average degree of asymmetry of the study population may have been a limitation because study groups that included patients with stronger mention deviation reported a significant influence of facial asymmetry on mandibular morphology [15–17].

### Generalizability of the results

When generalizing the study results, it must be considered that only adults with extreme malocclusions requiring orthognathic surgery were included. This facilitated the identification of correlations with specific mandibular characteristics. In the average population these differences might be less pronounced. However, it can be concluded that angular and volumetric measurements are more sufficient in discriminating sagittal and vertical malocclusions than linear measurements. Furthermore, looking at the ramus angle, gonial angle, body angle and body/mandibular volume might help the orthodontist to identify extreme skeletal patterns and to decide whether combined orthodontic and surgical treatment is required. Further studies based on our results should be performed.

The exact growth mechanisms causing the identified correlations can only be conjectured from the study results. As the mandible is the only movable bone of the craniofacial complex and is therefore constantly exposed to external influences [38], a strong effect by environmental and functional factors via epigenetic mechanisms is suspected [4, 39]. A well-investigated factor which contributes to mandibular morphology is the masseter muscle. Hypodivergent patients were reported to have greater volume [40], thickness [41] and masseter muscle activity [42] than hyperdivergent patients. As the masseter inserts at the angulus mandibulae, the ramus of hypodivergent patients is consequently exposed to higher forces, which might lead to the smaller gonial angle and due to an inhibition of the V principle [43] the identified smaller body angle. In class III patients the masseter muscle is more vertically orientated than in class II patients [44], which could cause the smaller ramal angle in class III patients compared to class II patients. In the next step, longitudinal 3D studies in adolescent study populations are necessary to clarify whether the observed differences in mandibular characteristics already appear during growth and can thus help clinicians to detect early tendencies towards severe malocclusions using 3D imaging.

The important role of the close link of form and function even after the end of growth was shown by studies in adult class III patients undergoing orthognathic surgery. They demonstrated increased activity and thickness of the masseter muscle 6–9 months after surgical correction [45–47]. Whether subsequently mandibular morphology also changes in these patients has not yet been investigated but should be focused on in future research.

## Conclusion

Based on a comprehensive 3D analysis, the present study revealed new important correlations between extreme skeletal patterns and mandibular characteristics in adults. It was demonstrated that especially angular and volumetric characteristics of a patient’s mandible could provide valuable hints about their sagittal and vertical growth pattern. These findings might help the clinician to decide on the need for orthognathic surgery. However, longitudinal studies based on our results are required.

### Supplementary Information

Supplemental Table 1: Intrarater and interrater reliability of all measurements assessed by Bland–Altman plots
