# Supplementary material for: Three-dimensional mandibular characteristics in skeletal malocclusion: A cross-sectional study
Source: J Orofac Orthop. 2022 Aug 26;85(2):134–45. doi: 10.1007/s00056-022-00419-1 (PMC10879264; doi:10.1007/s00056-022-00419-1)
Supplement: Supplementary file 1 — Supplemental Table 1: Intrarater and interrater reliability of all measurements assessed by Bland–Altman plots [file 56_2022_419_MOESM1_ESM.pdf]

Supplemental Table 1 Intra-rater and inter-rater reliability of all measurements assessed by Bland-Altman-plots

Zusätzliche Tabelle 1 Intra-Rater und Inter-Rater Reliabilität aller Messungen untersucht anhand von Bland-Altman-Diagrammen

**Characteristic**	**Intra-Rater**				**Inter-Rater**			
	M_{Diff}	SD_{Diff}	LoA-	LoA+	M_{Diff}	SD_{Diff}	LoA-	LoA+
Ramus height [mm]	1.1	1.2	-1.3	3.5	0.4	0.9	-1.3	2.1
Ramus width [mm]	0.2	0.4	-0.5	0.9	-0.3	0.4	-1.2	0.5
Body length [mm]	-0.3	1.2	-2.6	2.1	0.9	1.3	-1.6	3.4
Ramal angle [°]	0.2	0.9	-1.6	2.0	-0.1	1.1	-2.2	2.1
Gonial angle [°]	-0.3	1.1	-2.4	1.8	-1.3	1.2	-3.7	1.0
Body angle [°]	0.2	1.9	-3.5	3.8	-1.2	1.2	-3.5	1.1
Ramus/Mand volume [%]	0.0	0.4	-0.7	-0.8	-0.1	0.3	0.1	0.6
Body/Mand volume [%]	0.0	0.5	-1.1	1.0	0.1	0.4	0.2	0.5

Mand mandibular
